# Longitudinal changes of ocular blood flow using laser speckle flowgraphy during normal pregnancy

**DOI:** 10.1371/journal.pone.0173127

**Published:** 2017-03-03

**Authors:** Takahiro Sato, Junichi Sugawara, Naoko Aizawa, Noriyuki Iwama, Fumiaki Takahashi, Michiyo Nakamura-Kurakata, Masatoshi Saito, Takashi Sugiyama, Hiroshi Kunikata, Toru Nakazawa, Nobuo Yaegashi

**Affiliations:** 1 Department of Obstetrics and Gynecology, Tohoku University Graduate School of Medicine, Sendai, Miyagi, Japan; 2 Division of Feto-Maternal Medical Science, Tohoku Medical Megabank Organization, Tohoku University, Sendai, Miyagi, Japan; 3 Department of Ophthalmology, Tohoku University Graduate School of Medicine, Sendai, Miyagi, Japan; 4 Department of Obstetrics and Gynecology, Ehime University Graduate School of Medicine, Matsuyama, Ehime, Japan; University of North Carolina at Chapel Hill, UNITED STATES

## Abstract

**Purpose:**

Innovative laser speckle flowgraphy (LSFG) enables noninvasive evaluation of retinal microcirculation and the usefulness has been reported in the field of ophthalmology. LSFG has allowed us to measure the real time changes of retinal blood flow without pupillary dilatations and contrast media. Herein, we investigated the change of retinal blood flow in normal pregnant women during gestation using LSFG.

**Methods:**

A prospective cohort study was conducted in 53 pregnant women who visited our institution between January, 2013 and July, 2014. Finally, a total of 41 participants without any obstetric complications were available for evaluation. Retinal blood flow was measured with LSFG in a total of 4 times during pregnancy (T1. 11–13 weeks, T2. 19–21 weeks, T3. 28–30 weeks, T4. 34–36 weeks) and mean blur rate (MBR), blowout score (BOS), flow acceleration index (FAI), and resistive index (RI) are analyzed from these measurements. Relations between LSFG parameters and mean arterial blood pressure (MAP) are determined throughout pregnancy.

**Results:**

MBR showed no significant changes throughout pregnancy. BOS showed a tendency to increase in the 3rd trimester. FAI values showed a slight increase from the 1st to 2nd trimester while a significant decrease was noted in the 3rd trimester. RI exhibited no changes between the 1st and 2nd trimesters, values decreased significantly after the 3rd trimester. MAP was positively correlated with BOS, and negatively correlated with FAI and RI.

**Conclusion:**

The present study clearly demonstrated that profiles of LSFG parameters demonstrated a decrease of resistance in retinal blood vessels. These changes in indices provide a highly sensitive reflection of physiological changes in vascular resistance due to pregnancy. Thus, LSFG may be useful, as a non-invasive, diagnostic tool to detect pregnancy related disorders such as preeclampsia.

## Introduction

During normal pregnancies, blood pressure decreases during the 1st and 2nd trimesters as a result of increases in cardiac output and circulating plasma volume, increased peripheral vascular calibers due to dilation, and decreased peripheral vascular resistance. These are just some of the physiological changes that are known to occur in the cardiovascular system [1–3]. However, in pregnant women with preeclampsia and gestational diabetes mellitus, peripheral vascular resistance increases due to vascular endothelial damage. Early detection of these early-onset increases in vascular resistance would enable a clinician to predict the onset of obstetrical diseases and would lead an effective prevention and treatment of these conditions [4].

Several papers have been published on the physiological changes in fundus retinal vascular caliber [5]. A cohort study of patients with normal pregnancies reported that fundus imaging allowed observation of retinal vascular calibers over time and in the 2nd trimester of pregnancy, blood vessel caliber increases showed negative correlations with mean arterial pressures (MAP) [5]. Moreover, narrowing of retinal microvascular calibers has been reported to precede the onset of preeclampsia and increases in blood pressure [6].

In the past, a noninvasive ultrasound Doppler method was used to estimate peripheral vascular resistance and many papers have reported ocular artery blood flow wave changes in pregnancy [7–11]. Based on these reports, in normal pregnancy, ocular arterial blood flow resistance often decreases as the pregnancy progresses, and it has been pointed out that in preeclampsia, there are clear signs of abnormal blood flow. In this way, discussions of the related literature also show that while these methods may be effective as a means of analyzing the severity of the disease state and treatment efficacy, hardly any papers have looked at how these tests may be used to predict the onset of these complications [12,13].

Taken altogether, if real-time changes in fundus retinal blood flow changes could be determined objectively in microvasculature smaller than the ocular arteries using noninvasive methods, we believe more accurate detection of physiological changes in vascular resistance during pregnancy would be possible. Moreover, using this as reference data, we believe that by observing changes in blood flow, the onset of obstetric diseases that damage microvasculature could be predicted early on.

Laser speckle flowgraphy (LSFG) was recently developed as a new noninvasive method to evaluate fundus retinal blood flow in visible microvasculature and is commonly known as a fundus blood flow measurement device [14,15]. LSFG does not require the use of contrast agents or pupil dilation, and can assess 2-dimensional fundus blood flow indices quickly, leading to reports of its usefulness in the assessment of ocular disease states [16–18]. LSFG applies a laser to the blood flow and the scattering rays interfere with each other creating a random speckled pattern known as the speckle phenomenon. This technology takes advantage of this phenomenon and the faster the blood flow, the more the speckles move. Change rates in speckle patterns on LSFG can be expressed numerically and flow rate assessed as mean blur rate (MBR) and waveform analysis allow for measurement of blowout score (BOS) indicating that vascular resistance can be measured [19]. BOS allows measurement of the blood volume that pumps through vasculature during one heartbeat. BOS can be considered an index of vascular resistance, so it can be used to measure how much blood flow is maintained during a single heartbeat, and decreases in this value would indicate an increase in vascular resistance [19]. Flow acceleration index (FAI) allows calculation of the maximum magnitude of change in increasing MBR. It standardizes the explosiveness required to increase blood flow over a short period of time, with results expressed as the maximum change per frame (1/30th sec). Increases in these values reflect increases in vascular resistance. Resistive or resistance index (RI) is an indicator of the ultrasound Doppler method, and can be derived by dividing the difference between the maximum and minimum MBR values by the maximum value. Increases in RI indicate increased vascular resistance. Recently, these parameters have been shown to be highly useful because they are reproducible and allow for quantitation [20].

We investigated the retinal blood flow of optic nerve heads in pregnant women using LSFG for the first time in obstetrics and successfully analyzed the physiological changes that occur in retinal vasculature during pregnancy in a longitudinal study over time.

## Materials and methods

### Participants

A prospective cohort study was conducted in 53 pregnant women who visited the obstetrics department of Tohoku University between January 2013 and July, 2014, who gave their informed consent to participate in this study. Inclusion criteria included single pregnancies with no neonatal abnormalities, intrauterine growth retardation, smoking history, or complications of the cardiovascular, endocrine, or neurological system. Five women were excluded from the study during the course of their pregnancies. Another 7 developed complications of pregnancy-induced hypertension and finally, 41 participants were available for evaluation.

This study was conducted in compliance with the Declaration of Helsinki. Informed consent was obtained from all participants in writing, and the study was approved by the Tohoku University Institutional Review Board (2012-2-108-1).

### Laser speckle flowgraphy measurements

Details of the LSFG (Softcare, Fukutsu, Japan) and details regarding the principles of this technology was described in a previous paper [16,17]. The instruments included a diode laser (wavelength: 830 nm) attached to a fundus camera and an ordinary charge-coupled device camera (750 to 360 pixels). Relative blood flow rate was expressed in terms of MBR (mean blur rate: measured in arbitrary units: AU) and laser beam scatter interference by fundus circulation created a random speckle pattern which could be expressed numerically. MBR images taken consecutively at a rate of 30 frames per second over a 4-second measurement would be stored in a computer file. A blood flow map of the optic nerve head (ONH) would be created by synchronizing recorded MBR images with each cardiac cycle using analytical software.

**Fig 1:**
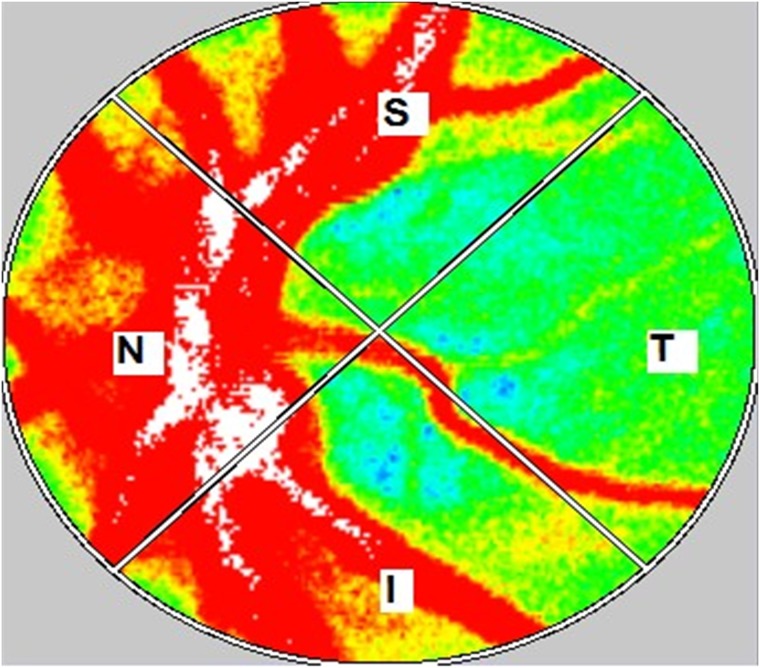
Representative color-coded map produced using LSFG. The colors in this map represent the time averages of MBR over one heartbeat. The elliptical area was set at the outer edge of the optic disk. Measurements were taken for the S: superior, T: temporal, I: inferior, and N: nasal quadrants.

Participants were measured a total of 4 times during pregnancy (T1. 11–13 weeks, T2. 19–21 weeks, T3. 28–30 weeks, T4. 34–36 weeks). LSFG of the left eye and blood pressure measurements were made after a 5-minute rest-period in all patients, and they were all examined by one experienced investigator. The mean arterial blood pressure was calculated using the formula MAP = DBP+1/3 (SBP–DBP), where DBP and SBP represent diastolic and systolic blood pressure levels, respectively. To determine intrasession reproducibility, LSFG was performed three times within 10 minutes. The margin of the ONH was identified manually, by placing an ellipsoidal band on the blood flow map (Fig 1). The position of the band was saved in software so that it could be used in subsequent scans of the same patient. Previous eye position was recorded using LSFG Measure (v 6.64.00; Softcare Ltd, Kyushu, Japan), which enabled us to capture the same area in the following examination. In addition, LSFG-NAVI allowed the investigator to adjust the focus by looking at the live-capture image.

The ‘‘vessel extraction” function of the software then automatically identified the vessel and tissue areas of the ONH, so that blood flow parameters could be assessed separately for the tissue area by itself (referred to as MT, ‘‘mean value tissue area”) and the total area of the ONH (MA, ‘‘mean value all areas”). Next, we calculated the following 3 waveform analysis parameters: blowout score (BOS), flow acceleration index (FAI), and resistive index (RI) [19]. Finally, we analyzed correlations between mean arterial pressure (MAP) and parameters of LSFG throughout pregnancy.

### Statistical analysis

The means of 3 LSFG measurements were used in the statistical analysis. Analysis of changes in parameters during pregnancy were corrected for age and BMI to fit into a generalized linear-mixed effect model to assess the longitudinal trend of data. This model was conducted with inclusion of intersubject effects and timepoint as repeated measurements, the specific LSFG parameters as dependent variables. Pearson correlation coefficients were used to assess relationships between MAP and each measurement variable (i.e. MBR, BOS, FAI and RI). SAS Ver. 9.4 Software (SAS Institute Japan Inc., Tokyo, Japan) was used for the statistical analysis. If no other indications are given, then the data are expressed as mean ± standard error (S.E.), and a P value of <0.05 is considered statistically significant.

## Results

**Fig 2:**
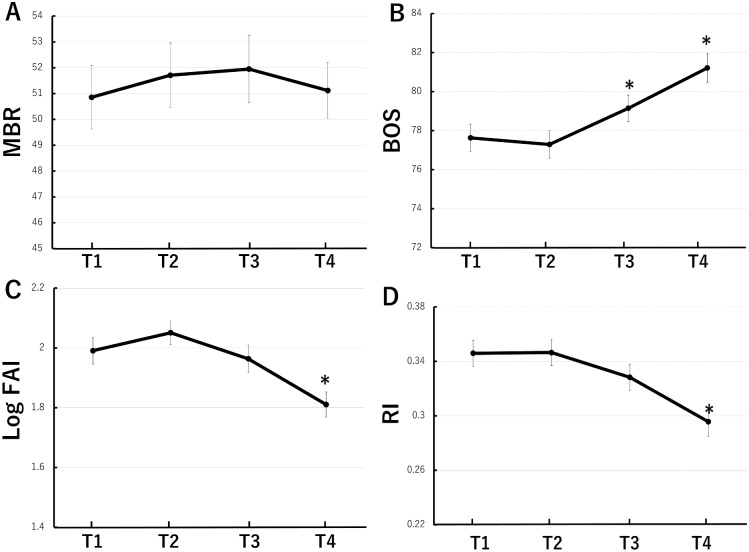
Longitudinal changes of LSFG parameters throughout pregnancy. Assessment of LSFG measurements at each time point (T1. 11–13 weeks, T2. 19–21 weeks, T3. 28–30 weeks, T4. 34–36 weeks) for MBR (A), BOS (B), FAI (C), and RI (D). Generalised linear mixed model was used to determine level of significance versus T1. * = p<0.05. MBR: mean blur rate, BOS: Blowout score, FAI: Flow acceleration index, RI: Resistive index.

Clinical profiles of the 41 patients analyzed were ages: 33.3±5.9 years, systolic blood pressure(SBP): 111.7±10.2, diastolic blood pressure (DBP): 69.8±9.6, BMIs before pregnancy: 21.8±4.5, weeks of gestation: 38.7±1.3, neonatal body weights: 2957±400.4 g (Table 1). Profiles of LSFG parameters over the course of the pregnancy are shown in Fig 2. No apparent changes in MBR were noted over the course of the pregnancies T1. 50.9±7.9, T2. 51.7±8.1, T3. 51.9±8.4, T4. 51.1±7.0 (Fig 2A). BOS values were: T1.77.6±4.6, T2.77.3±4.6, T3.79.1±4.4, T4.81.2±4.8, and although no changes were noted from the T1 to T2, in T3 and T4, increases were noted as the pregnancy progressed (Fig 2B). FAI values were T1. 7.6±2.2, T2. 8.1±2.0, T3. 7.4±2.1, T4. 6.3±1.8, and there was a slight increase from T1 to T2 while a significant decrease was noted in T4 (Fig 2C). RI was T1. 0.346±0.062, T2. 0.346±0.063, T3. 0.328±0.062, T4. 0.296±0.070, and although no changes were noted between T1 and T2, values decreased significantly in T4 (Fig 2D).

**Table 1:** Baseline characteristics of participants.

Characteristics	Values
No. of subjects	41
Maternal characteristics	
Age (years)	33.3(5.9)
<30 years (%)	40.0
30–34.9 years (%)	22.5
≥35 years (%)	37.5
Height (cm)	159.8 (5.9)
Pre-pregnancy weight (kg)	56.0 (12.0)
Pre-pregnancy BMI (kg/m^2^)	21.8 (4.5)
Gestational weight gain (kg) (n = 40)	8.9 (3.1)
SBP (mmHg)	111.7 (10.2)
DBP (mmHg)	69.8 (9.6)
Primipara (%)	43.9
Family history of hypertension (%)	58.3
Family history of diabetes mellitus (%)	19.5
Gestational age at first LSFG, week (Median, IQR)	12.0, 11.8–12.5
Gestational age at second LSFG, week (Median, IQR)	20.1, 19.9–20.9
Gestational age at third LSFG, week (Median, IQR)	29.1, 28.4–29.6
Gestational age at fourth LSFG, week (Median, IQR)	36.2, 35.2–36.6
Neonatal characteristics	
Sex (male/female) (%)	53.5 / 46.5
Gestational age at delivery, week	38.7 (1.3)
Birth weight, g	2957 (400)

Values are means±standard error. BMI = body mass index, SBP = systolic blood pressure, DBP = diastolic blood pressure. IQR = interquartile range

**Fig 3:**
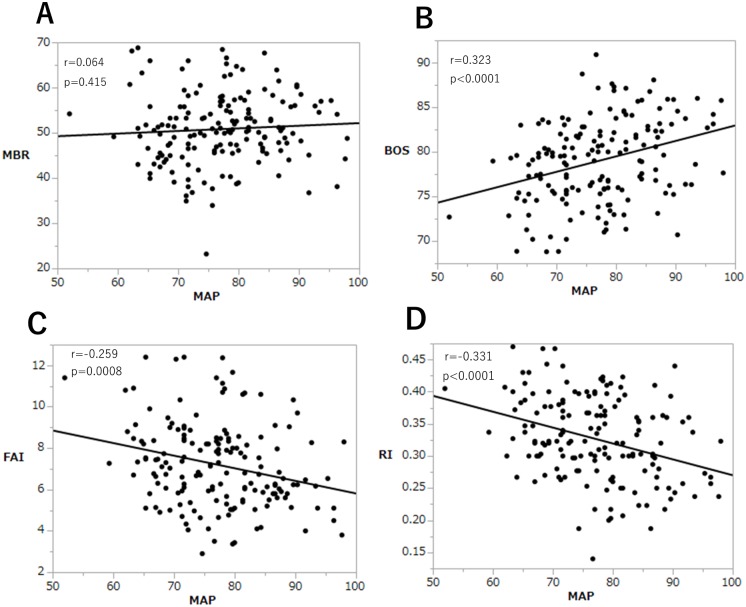
Relationship between MAP and LSFG parameters. Correlation between MAP and LSFG measurements were analyzed for MBR (A), BOS (B), FAI (C), and RI (D). Pearson correlation coefficients were used to assess relationships between MAP and each measurement variable. MAP: mean arterial pressure, MBR: mean blur rate, BOS: Blowout score, FAI: Flow acceleration index, RI: Resistive index.

Next, we analyzed correlations between MBP and LSFG parameters, MBR, BOS, FAI and RI. There are no correlations between MAP and MBR throughout pregnancy (r = 0.064, p = 0.415, Fig 3A). Interestingly, there was a significant trend towards increased BOS (r = 0.323, p<0.0001) and decreased FAI (r = 0.259, p = 0.0008) and RI (r = 0.331, p<0.0001) with MAP (Fig 3B, 3C and 3D).

## Discussion

In this study, the recently developed LSFG allows longitudinal measurements of pregnancy-related changes in optic nerve head circulation in a noninvasive manner. BOS, an index of LSFG blood flow, showed a tendency to increase in the 3rd trimester (T3 and T4), while FAI and RI values decreased significantly in the 3rd trimester. Furthermore, BOS was positively correlated with MAP, whereas FAI and RI showed negative correlations with MAP throughout pregnancy. These changes in indices provide a highly sensitive reflection of physiological decreases in vascular resistance due to pregnancy.

Retinal vasculature self-regulates blood flow in response to various physiological stimuli, and this is adjusted by ocular perfusion pressure and vascular resistance [21]. Changes in vascular resistance are generally influenced by vascular endothelial function and vascular smooth muscle elasticity, but in pregnancy, decreases in vascular resistance are attributed to activation of vascular endothelial function. Results of monitoring blood flow indices by LSFG in this study match what has been reported in previous studies that have used ultrasound Doppler methods to show a decrease in RI during progression of a normal pregnancy [8,11]. Belfort's report states that in normal pregnancy, the cerebral and orbital RI and MAP are negatively correlated [8]. Kyle et al reported that increasing MAP by angiotensin infusion resulted in a reduction of RI during the period of increased blood pressure [22]. From these observations, explanations for why the cerebral resistance may decrease with the increase in MAP is that this condition tends to shunt blood away from cerebral circulation, and as a physiological adaptation, cerebral blood flow is maintained by an autoregulatory vasodilation [8]. Taken together, the results from our study suggest that RI using LSFG are strongly associated with changes of MAP.

Until now, the objective was to identify changes in the ocular artery resistance in obstetric disease, and many studies used ultrasound Doppler methods, where clear signs of change were noted in preeclampsia [4]. Recent reports state that narrowing of retinal vascular calibers in preeclampsia have been observed to precede any increases in blood pressure [6]. This suggests that changes in vascular resistance due to changes in retinal microvascular caliber may be seen before any changes in blood pressure become apparent. Monitoring blood flow index changes during a normal pregnancy by LSFG in this study will provide a vital reference in the analysis of obstetric diseases such as pregnancy-induced hypertension and gestational diabetes.

The limitation of this study is that since informed consent to participate in this study was obtained from pregnant women who visited the outpatient obstetrics clinic, a longitudinal analysis of these patients from before pregnancy was not possible and so it was difficult to assess any individual changes in blood flow during the first trimester. The limited sample sizes in cohort studies makes it difficult to analyze disease prediction in conditions such as preeclampsia. Hereafter, in order to overcome these issues, we plan to expand the cohort study to better allow us to analyze the relationship between the onset of various obstetric diseases and their pathophysiology.

In conclusion, this study shows for the first time that the recently developed LSFG can be used in longitudinal observations of blood flow changes in the visual nerve head thus allowing sensitive detection of changes in peripheral vascular resistance in the field of obstetrics. LSFG is a simple, fast, noninvasive diagnostic tool with a high level of reproducibility, and may prove broadly valuable when applied in the early discovery and pathophysiological assessment of obstetric diseases such as preeclampsia.
